# Long-term positioning and polar preference of chemoreceptor clusters in *E. coli*

**DOI:** 10.1038/s41467-018-06835-5

**Published:** 2018-10-25

**Authors:** Moriah Koler, Eliran Peretz, Chetan Aditya, Thomas S. Shimizu, Ady Vaknin

**Affiliations:** 1 The Racah Institute of Physics, The Hebrew University, Jerusalem, 91904 Israel; 2 AMOLF Institute, Amsterdam, 1098XG The Netherlands

## Abstract

The bacterial chemosensory arrays are a notable model for studying the basic principles of receptor clustering and cellular organization. Here, we provide a new perspective regarding the long-term dynamics of these clusters in growing *E. coli* cells. We demonstrate that pre-existing lateral clusters tend to avoid translocation to pole regions and, therefore, continually shuttle between the cell poles for many generations while being static relative to the local cell-wall matrix. We also show that the polar preference of clusters results fundamentally from reduced clustering efficiency in the lateral region, rather than a developmental-like progression of clusters. Furthermore, polar preference is surprisingly robust to structural alterations designed to probe preference due to curvature sorting, perturbing the cell envelope physiology affects the cluster-size distribution, and the size-dependent mobility of receptor complexes differs between polar and lateral regions. Thus, distinct envelope physiology in the polar and lateral cell regions may contribute to polar preference.

## Abstract (web summary)

Bacterial chemoreceptors form clusters, preferably at  the cell poles. Here, Koler et al. show that polar and lateral clusters exhibit distinct long-term positional dynamics   and that polar bias may be due to differences in mobility of receptor complexes between the polar and lateral cell regions.

## Introduction

As more elements of the bacterial cell are found to exhibit a unique static or dynamic spatial distribution, the underlying mechanisms that control these phenomena are progressively being revealed^1,2^. *E. coli* chemoreceptors were among the first membrane-bound bacterial proteins that were shown to form large clusters with a clear polar preference^3,4^, and such chemosensory clusters were later found in many other motile bacteria^5^. However, while the organization of chemoreceptors within clusters is becoming better understood, the dynamics leading to their formation and the mechanisms that control their positions in cells are not clear.

**Fig. 1:**
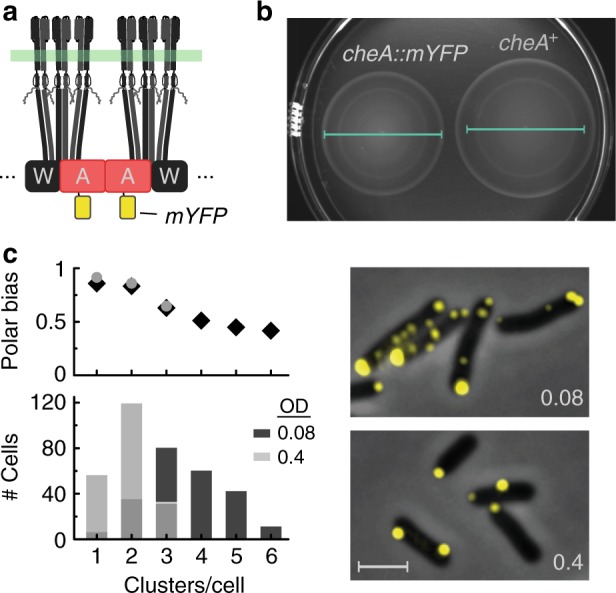
Basic characterization of the MG1655/*cheA::mYFP* (MK4) cells. **a** Schematic description of core complexes showing the position of the mYFP tag in the core complex. **b** Colony expansion of the *MG1655* (CheA^+^) cells and the *cheA::mYFP* derivative in soft agar chemotaxis plates after 10 h at 30 °C. Bars are of the same size. **c** Fluorescence images of *cheA::mYFP* cells grown to an optical density (OD_600_) of 0.08 or 0.4 in liquid culture. Scale bar corresponds to 2 µm. Histogram of the number of clusters per cell in the two populations (240 and 209 cells, respectively) and the respective polar bias of the clusters (number of polar clusters / total number of clusters) in each subpopulation of cells belonging to each bin of the histogram are also shown

The chemosensory clusters contain up to several thousand receptors that modulate the activity of an associated histidine kinase and ultimately control the swimming behavior of the bacterium^6^. The rod-shaped bacterium *E. coli* has five types of chemoreceptors with different sensory specificities that form mixed core signaling complexes. Each core complex contains two receptor heterotrimers of homodimers bound to a dimeric CheA kinase and two CheW linker proteins (Fig. 1a)^7^. These complexes form extended arrays through binding interactions between the linker protein CheW and the P5 domain of the kinase CheA^8–10^. Receptor clustering generally leads to high cooperativity in the kinase control and to signal amplification^11–15^, mostly through allosteric coupling between core complexes^8,16^, that ultimately leads to efficient and robust chemotaxis^17^.

The distributions of chemoreceptor cluster sizes and the distances between them have led to the suggestion that receptor clustering occurs via free diffusion and capture^18–20^. The preference of clusters toward the cell poles was suggested to result from the fact that as cells grow and divide, mid-cell clusters can become polar^4,18,20,21^. On the other hand, other studies suggested that the positioning of chemoreceptor clusters in *E. coli* is directly driven by various factors, including membrane curvature^22–24^, direct interactions with the Tol–Pal system^25^, and nucleoid occlusion^26^. The abundance of cardiolipins in polar regions may also contribute to polar bias but does not appear to play a major role in *E. coli* cells^25^.

In this study, by monitoring growing cells with fluorescently tagged receptor clusters for up to 6 h (approximately nine generations), we provide a long-term perspective regarding the cluster dynamics in *E. coli*. We demonstrate that lateral clusters tend to avoid translocation to the new cell pole regions after cell division and thus keep shuttling between the cell poles. Quantitative analysis of their positional dynamics indicates that lateral clusters tend to be static relative to the local cell-wall matrix for many generations, at least, along the long axis of the cell. Overall, the long-term positional dynamic of a cluster is predominantly determined by whether it was initially nucleated within the lateral cell envelope region, which undergoes elongation, or the polar cell envelope region, which is generally inert. We also show that the polar preference of these clusters results from intrinsically reduced clustering efficiency in the lateral cell region. Surprisingly, polar bias of receptor complexes is evident regardless of the structural integrity of receptor arrays, the length and flexibility of receptor dimers, or the location of the contacts between receptors, challenging the notion that the specific structural properties of receptor complexes promote their polar preference due to sorting by membrane-curvature affinity. On the other hand, chloramphenicol, which leads to nucleoid condensation, and TolA, which connects the cytoplasmic membrane with the cell-wall matrix, can affect receptor clustering. Notably, by their nature, such effectors can lead to distinct local environments in the polar and lateral regions. We indeed find that the manner in which the mobility of receptor complexes depends on their size differs between the polar and lateral regions, which can potentially contribute to the observed polar bias.

## Results

### Long-term positioning of chemoreceptor arrays

To study the positioning of chemoreceptor clusters, we constructed a *cheA::mYFP* variant of the *E. coli* MG1655 strain (MK4) containing a chromosomal insert of a monomeric mYFP(A206K) tag^27^ between the P1 and P2 domains of CheA (Fig. 1a). This strain exhibited nearly normal chemotaxis behavior in soft agar plates^28^ (80–90% of the wild type; Fig. 1b). The tagged CheA did not cluster in a strain lacking the chemotaxis receptors, but integrated into clusters promoted by the native receptors (Fig. 1c). The distribution of the number of detectable clusters per cell at two growth stages of the culture (OD 0.08 or 0.4) and the polar bias in each bin is shown in Fig. 1c. Notably, lateral clusters, which were often observed by various methods^3,20,21,29^, were more common during the early growth stage of the culture and, correspondingly, the averaged polar bias was lower during these stages.

To gain a long-term perspective regarding clusters positioning, we spread the cells on the surface of an agarose gel at a low concentration and starting from a well-isolated single cell followed the receptor clusters in the growing colony for up to 6 h (Methods section). We estimate that the smallest cluster that could be reliably identified under these conditions corresponds to ~20–30 fluorophores or 10–15 core complexes (Supplementary Fig. 1). In some experiments, we also followed the dynamics of the *Z*-ring by expressing mCherry-tagged FtsZ proteins in addition to the natively expressed FtsZ proteins^30^.

**Fig. 2:**
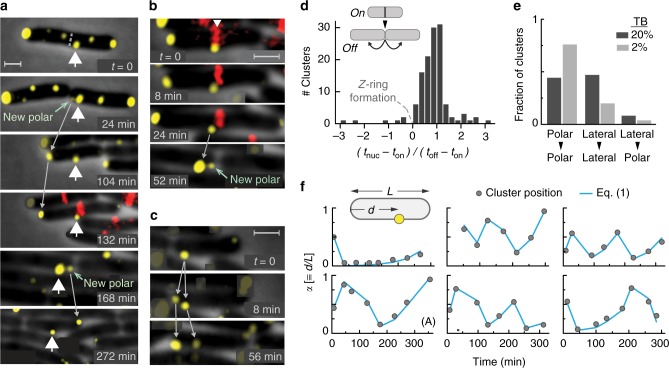
Typical long-term positional dynamics of clusters in the *cheA::mYFP* (MK4) cells. **a** In this example, cells also expressed FtsZ-mCherry (induced by 0.005% arabinose); however, for clarity, the mCherry overlay was added only at the 132 min time point. The central cluster tracked here is labeled throughout the time series by a thick white arrowhead. The new clusters that appeared near the central cluster in the cell pole at 24 and 168 min time points are also labeled. For clarity, few of the high intensity clusters in cells other than those in focus were masked. Fluorescence images were recorded every 4 min, and only sample images are shown. **b** Similar to **a** with a focus on a single division event. **c** Example of a cluster near the cell pole that apparently split into two with one cluster remaining at the pole and the other drifting away. Scale bars corresponds to 1 µm. **d** Histogram of polar-cluster nucleation times (*t*_nuc_) measured relative to the period between the assembly (*t*_on_) and disassembly (*t*_off_) of the *Z*-ring during the corresponding cell division (127 clusters). **e** Using cells without FtsZ-mCherry, clusters within a certain colony at a certain time point were identified and grouped according to their nucleation and final positions, polar or lateral. The data are shown for cells grown in minimal medium containing 20% (dark gray) or 2% (light gray) TB (301 and 133 clusters, respectively). **f** The relative position of the clusters (*α*) was defined as the distance between the cluster and a certain cell pole normalized by the length of the cell and is plotted as a function of time (gray circles). Each plot represents the trajectory of a single cluster whose position was quantitatively evaluated once every cell cycle, soon after cell division. The estimated uncertainty in the measured cluster relative position is approximately the size of the symbols. The blue lines were plotted by iterating equation (1). In total, 22 clusters from nine independent experiments were followed, each for several generations (see also Supplementary Fig. 3)

Typical positional dynamics of clusters is demonstrated in Fig. 2a–c (see also Supplementary Fig. 2). At the beginning of the timeline shown in Fig. 2a (*t* = 0), the labeled cluster (white arrow) was located close to the future division site, and after cell division (24 min), the labeled cluster was close to the cell pole. However, the original cluster drifted from the pole during the division process, and a new polar cluster nucleated at the newly formed pole. As the cell kept growing (104 min), the new polar cluster remained at the pole region, but the original cluster (white arrow) drifted further, toward the middle of the cell. As the next division approached, the *Z*-ring assembled very close to the original cluster (132 min; red label/white arrow). However, the original cluster (white arrow) was again displaced from the division site during cell division (168 min), and a new polar cluster nucleated at the newly formed cell pole. Then, again, the new polar cluster remained in the pole region while the original cluster (white arrow) again drifted toward the middle of the cell. As shown in Fig. 2b, even in cases where the pre-existing mid-cell cluster was directly positioned at the future division site marked by the *Z*-ring, the *Z*-ring eventually assembled next to the cluster, and after cell division the original mid-cell cluster (white arrow) drifted away from the cell pole and a  new cluster nucleated directly in the pole region. We could also identify a few cases in which a cluster near the boundary between the pole and lateral regions effectively split into two clusters that then exhibited clearly distinct long-term behaviors: the cluster closer to the cell tip remained in the pole, while the cluster that was slightly away from the pole drifted away from the pole (Fig. 2c and Supplementary Fig. 2C).

To obtain a more global view of the positional behaviors of the clusters in these cell populations, we studied the relationship between the nucleation of the clusters and their long-term fate in two ways. We first analyzed the nucleation of polar clusters with respect to the progression of cell division (Fig. 2d). This analysis was performed by using the assembly of the *Z*-ring as a marker of the beginning of cell division, time *t*_on_, and the dissociation of the *Z*-ring as a marker of the end of the constriction process, time *t*_off_ (see illustration in Fig. 2d)^31^. For each cell division event, we followed the two new cell poles that had been created and searched for clusters in these regions that remained polar in future generations; the nucleation times, *t*_nuc_, of these clusters were determined, and their relative nucleation times, *t*_rel_ ≡ (*t*_nuc_ *−* *t*_on_)/(*t*_off_ − *t*_on_), were calculated. We analyzed in this way cell populations grown in 2 or 20% TB. The point of nucleation can be generally ambiguous; here, the nucleation point is practically defined as the point where the receptor complex was large enough to become visible (Supplementary Fig. 1). Consistent with the examples shown in Fig. 2a–c, the histogram of nucleation times, shown in Fig. 2d, indeed demonstrates that the vast majority of polar clusters that remained polar in future generations nucleated after the assembly of the *Z*-ring (*t*_rel_ > 0) and only a small fraction of polar clusters was clearly visible before the formation of the *Z*-ring (*t*_rel_ < 0).

We also analyzed the positional behaviors of the clusters in cells lacking the FtsZ tag. In this case, we chose a certain frame near the middle of the timeline and identified all the clusters in the colony. We then traced the nucleation event and final position of each cluster within that frame and classified them into the following three groups according to their initial and final positions: lateral–lateral, polar–polar, and lateral–polar, respectively (Fig. 2e). We did not observe clusters that nucleated at a pole and became lateral. Cluster nucleation was considered ‘polar’ if it occurred during or after cell division. Since the *Z*-ring could be observed in the cells with tagged FtsZ before cell constriction could be clearly identified in phase contrast, a minority of polar clusters in the current analysis (<10%) that nucleated one or two frames before cell constriction could be clearly identified in phase contrast was still considered as correlated with the division process and, thus, ‘polar’ borne. The results of this analysis with cells grown in 2 or 20% TB are shown in Fig. 2e. Consistent again with the examples shown in Fig. 2a–c, under both growth conditions, the clusters that nucleated at the cell pole during or after cell division clearly remained at the pole, and the clusters that nucleated at the lateral region tended to avoid translocating to the pole regions and effectively shuttled between the cell poles.

We further analyzed the positional dynamics of the lateral clusters by following their relative position along the cell, *α*, defined as *α* = *d/L*, where *d* is the distance between the cluster and a reference cell pole (left or right; chosen arbitrarily, but consistently across generations), and *L* is the length of the cell (Fig. 2f). As the cells grow and divide, the position of a certain cluster was followed for several generations and quantitatively determined once every cell cycle soon after cell division (Fig. 2f and Supplementary Fig. 3). The long-term positional dynamics of clusters could be quantitatively explained by the following two assumptions: (i) clusters are fixed relative to their local cell-wall environment, at least, along the long axis of the cell, and (ii) throughout the time course, lateral clusters remain within the dynamic region of the cell envelope that undergoes elongation. Under these conditions, the relative position of a cluster after the *n* cycle of elongation and division is expected to follow the iterative proration given by (Supplementary Note 1),

1 $$\alpha _n = 2 \cdot \alpha _{n - 1}\,{\rm {modulo}}\,1.$$

The blue lines in Fig. 2f and Supplementary Fig. 3 were obtained by iterating Eq. (1), starting from an initial value constrained by the first measured value and, thus, with essentially no free parameters. Clearly, the positional dynamics of these clusters is well described by Eq. (1).

### Polar preference of chemoreceptor arrays

**Fig. 3:**
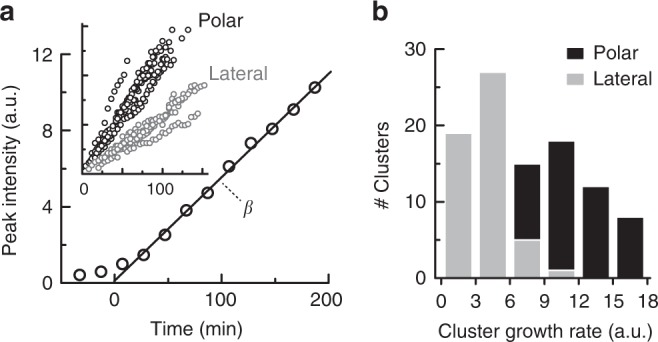
Initial cluster growth rate. **a** The main plot presents the growth of a polar cluster, as assessed by following the peak fluorescence intensity. The ‘time-zero’ point was defined by extrapolating from the apparent linear regime to zero intensity (black line). The cluster growth rate (β) was defined as the slope of the growth curve at the linear regime. The inset shows additional examples of polar and lateral clusters. **b** Histogram of the cluster growth rates (β) of polar (black) and lateral (gray) clusters (105 clusters)

Given that the combined surface area of the two cell poles is considerably smaller than the lateral surface area, the presence of even a similar number of polar and lateral clusters (Fig. 1c and Supplementary Fig. 2E) represents a significant polar bias in the nucleation probability per unit area. Moreover, the polar clustering preference manifested not only in the positioning of the clusters but also in their growth dynamics (Fig. 3). The growth of the clusters, as estimated by following the peak fluorescence intensity, seems to reach a regime in which the growth rate was approximately constant (Fig. 3a). Several examples of the growth curves of polar and lateral clusters are shown in the inset in Fig. 3a, and a histogram of their growth rate (β) is shown in Fig. 3b. The polar clusters clearly grew faster than the lateral clusters.

**Fig. 4:**
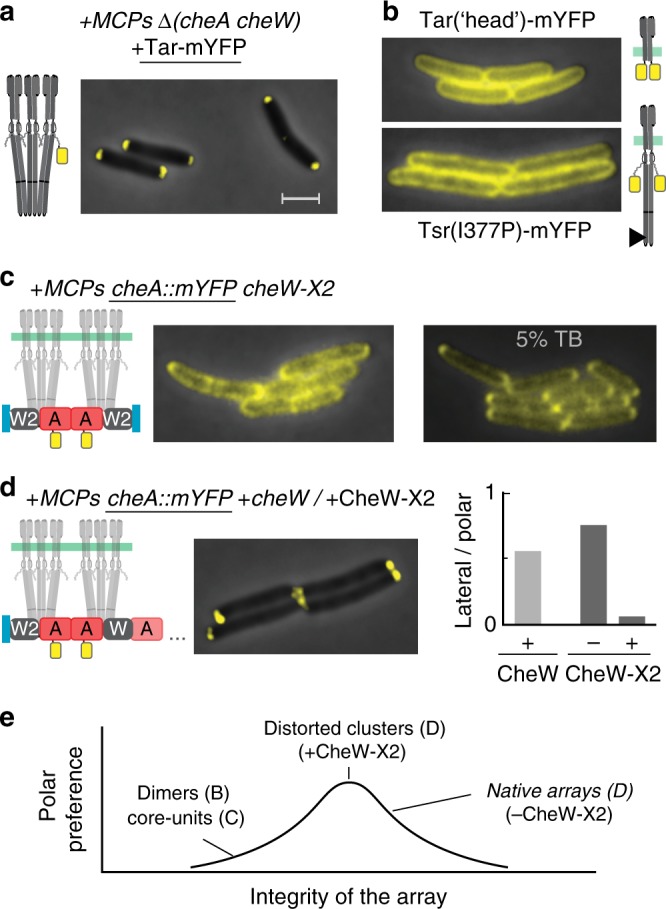
Positioning of modified receptor clusters. **a** Tar-mYFP expressed in addition to the native receptors in cells lacking CheA and CheW (UU1607). **b** Tar(head)-mYFP or Tsr(I377P)-mYFP expressed as the sole chemoreceptor in cells lacking CheA and CheW (UU2806). **c** *cheA::mYFP cheW-X2* cells (MK9) grown under standard conditions (left) or slow-growth conditions (5% TB, right). **d** Image: *cheA::mYFP* cells (MK4) expressing additional CheW-X2 proteins (pAV305, induced by 1 µM NaSal). Bar graph: The fraction of lateral clusters in MK4 cells with or without the addition of CheW-X2 (dark gray; 0 or 1 µM NaSal) or MK4 cells with additional CheW expressed from an analogous plasmid (light gray; 1 µM NaSal). See also Supplementary Fig. 4. **e** A schematic summary (see text). Scale bar corresponds to 2 µm, throughout

We then tested how the structural properties of chemoreceptor arrays affect their polar bias. Chemoreceptor trimers of dimers are formed through direct binding interactions between dimers at their cytoplasmic tips^32^. In the absence of CheA/W, direct interactions between receptor dimers can lead to the formation of larger complexes and even apparent clusters^33^. Indeed, when Tar-mYFP was expressed in cells containing all the native (untagged) receptors but deleted for the *cheA* and *cheW* genes, we observed clear polar clustering bias (Fig. 4a and Supplementary Fig. 4A). However, weakening the direct interactions between dimers by either deleting most of their signaling domain or by introducing the I377P mutation close to their contact region^32^ clearly led to a nearly uniform distribution of these receptors (Fig. 4b). Thus, the polar bias of the receptors in the absence of CheA and CheW is directly promoted by their clustering.

An additional way of preventing the formation of large receptor arrays but maintaining the receptor trimer structure and core-complexes is introducing the CheW-X2 mutation, which weakens the interactions between core complexes^8,17^. The binding of CheA and CheW-X2 to receptor trimers can block their direct associations while the CheW-X2 mutation prevent the association between core complexes. Similar to the observations reported in refs. ^8^^,^^17^, introducing the *cheW-X2* mutation to the chromosome of the *cheA::mYFP* strain (MK9) led to a nearly homogenous distribution of the label throughout the cell membrane (Fig. 4c), indicating that core complexes do not have an appreciated preference toward the cell pole. However, in cells grown in a poorer medium (5% TB), a clear polar preference was observed in these *cheW-X2* cells (Fig. 4c), which might suggest that under such slow-growth conditions small complexes like core-complexes already have a notable polar preference. Alternatively, given the strong polar bias observed in the absence of CheA/W (Fig. 4a), it is possible that under slow-growth conditions the ‘screening’ of the direct interactions between the receptors by binding of CheA/CheW-X2 is less efficient and, thus, leading to formation of receptor complexes larger than a single core-complexes.

Interestingly, while the *cheW-X2* cells (MK9) exhibited a reduced polar bias (Fig. 4c), expressing CheW-X2 in addition to the native CheW substantially diminished the lateral clusters and, therefore, considerably enhanced the polar clustering bias (Fig. 4d and Supplementary Fig. 4B). Only a moderate reduction in lateral clustering was observed when native CheW was  additionally expressed in the same cells (Fig. 4d), suggesting that the effect of excess CheW-X2 is primarily due to the introduction of interface 2 defects to the receptor arrays rather than merely the over-expression of CheW. The tolerance of the receptor array structure to a few fold excess CheW has been recently noted by Piñas and Parkinson (personal communication). Thus, a cohesive array structure is not essential for polar preference but, instead, weakening the arrays tends to enhance their polar preference. Overall, receptors polarity is basically driven by the formation of receptor complexes and clustering, but a cohesive array structure tend to enhance lateral clustering and, thus, reduces the polar preference (Fig. 4e).

**Fig. 5:**
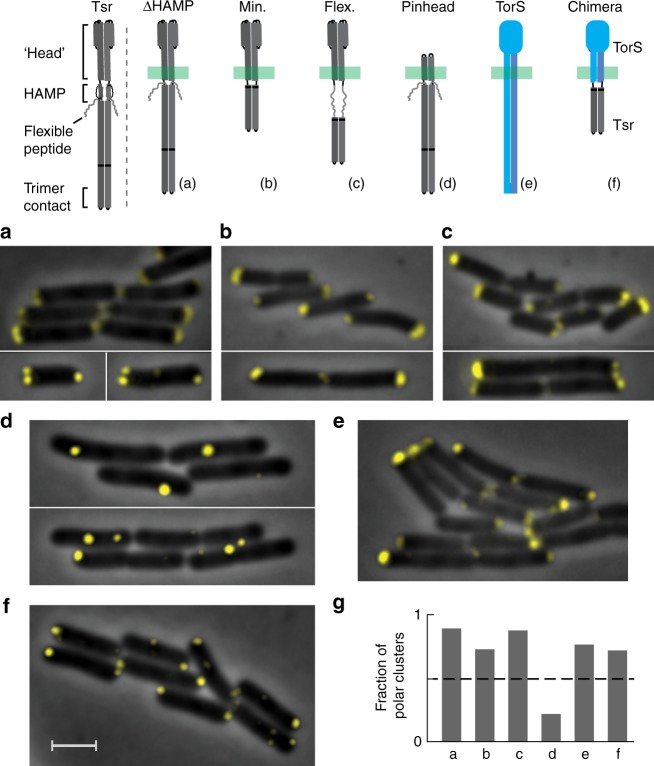
Positioning of modified receptors. A schematic description of the receptor variants is provided in the upper panel (see also Supplementary Fig. 5). **a**–**d** and **f** Fluorescence images of Δ(*cheA cheW MCPs*) (UU2806) cells expressing various receptor mutants from an inducible plasmid (pRR48, 100–200 µM IPTG) together with CheA::mYFP and CheW (pAV295,  0.3 µM NaSal). **e** Images of TorS-mYFP expressed in MG1655 cells (pES42, induced with 10 µM IPTG). **g** The fraction of polar clusters found in cells expressing the various receptor variants (a total of 208, 148, 88, 206, 169, and 191 clusters analyzed in parts **a**–**f**, respectively). Notably, with the exception of part **d**, the overall polar bias of these receptor variants is significantly larger since the clusters were generically larger in the pole regions than in the lateral region. Additional images are presented in Supplementary Fig. 6. Scale bar corresponds to 2 µm, throughout

Subsequently, we tested how the structural properties of the receptors affect their polar bias by testing modified receptor constructs (Fig. 5, top panel; Supplementary Fig. 5 and Table 1). To eliminate possible rigidity in the relative orientations of the signaling and transmembrane domains, we constructed Tsr(∆HAMP) where the HAMP domain was replaced with a glycine linker. Tsr(min) was constructed from Tsr(∆HAMP) by removing most of the Tsr signaling domain, leaving only the cytoplasmic tip (346–430) connected to the transmembrane domain. Tsr(flex) was constructed from Tsr(min) by inserting the C-terminal flexible peptide (520–546) between the transmembrane and the receptor tip domains. All these versions of Tsr recruited CheA::mYFP and formed clusters with a clear polar preference (Fig. 5a–c and Supplementary Fig. 6). A clear polar preference was also previously observed with Tsr receptors that were longer than the native Tsr^34^ (Supplementary Fig. 6G). Tsr(pinhead) was constructed from Tsr(∆HAMP) by replacing the periplasmic-transmembrane (‘head’) domain with a Tar head domain missing most of the periplasmic ligand-binding domain^35^. These receptors recruited CheA::mYFP and formed clusters but showed a significantly reduced polar preference (Fig. 5d). TorS is a transmembrane sensory histidine kinase that has been previously shown to form clusters through interactions between the head domains^36^. Nevertheless, the tagged TorS receptors also exhibited a clear polar bias (Fig. 5e and Supplementary Fig. 6). Moreover, fusing the TorS head domain, which could mediate clustering on its own^36^, with the Tsr tip domain (346–430) still recruited CheA::mYFP and formed clusters with a clear polar preference (Fig. 5f and Supplementary Fig. 6). Overall, the polar clustering bias did not depend on the length of the receptors, their rigidity, or even the locations where they contacted each other. The reason for the reduced polar preference of the Tsr(pinhead) receptors is not clear; interactions of the head domains in the periplasmic space might affect their clustering, but the missing domain might also impose critical constraints on the cytoplasmic or transmembrane domains and possibly affect the mobility of these constructs in the membrane.

### The membrane environment affects receptor clustering

**Fig. 6:**
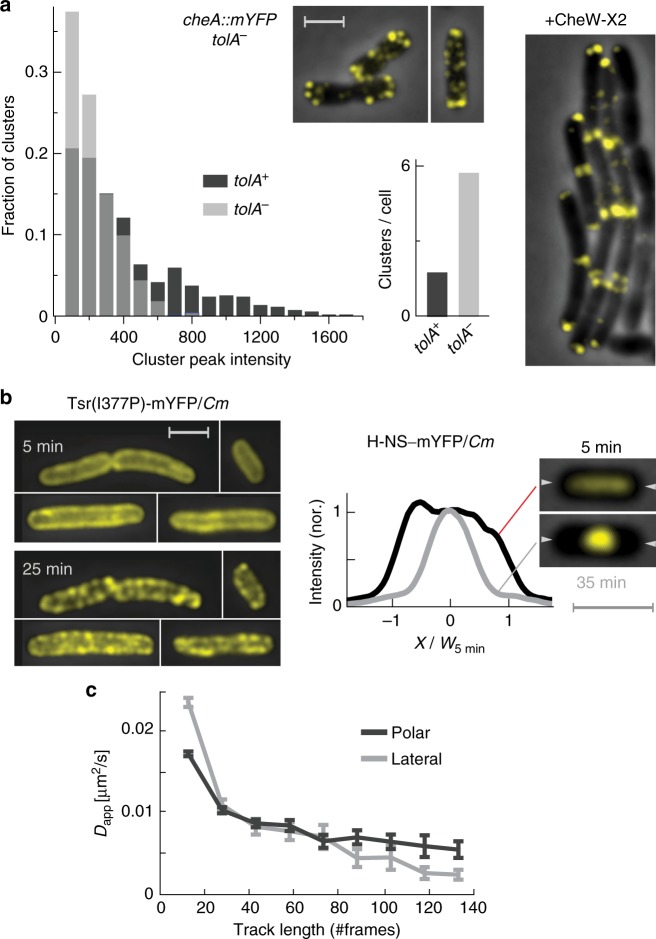
The effect of altered membrane environments on receptor clusters. **a** Effect of TolA. Distribution of cluster intensity in *cheA::mYFP* cells with (MK4; dark gray) or without (MK13; light gray) TolA. A total of 472 clusters (in 264 cells) or 475 clusters (in 82 cells) were sampled from each strain, respectively. Fluorescence images of typical TolA^−^ cell is also shown. The fluorescence image on the right demonstrate polar bias in TolA^−^ cells in which CheW-X2 was also expressed from a plasmid using 1 µM NaSal (see also Supplementary Fig. 7). **b** *Left: Δ(cheA* *cheW* *MCPs*) (UU2806) cells expressing Tsr(I377P)-mYFP receptors and imaged at various times after exposure to *Cm* (20 µg/ml). *Right:* UU2806 cells expressing H-NS-mYFP (pAV357, induced by 2 µM IPTG) and imaged at various times after exposure to *Cm* (20 µg/ml). Corresponding intensity profiles measured along the long axis of the cell (between the marks) are also shown. See also Supplementary Fig. 8. Scale bars correspond to 2 µm. **c** Mobility of receptor complexes in the polar (dark gray) and lateral (light gray) cell regions measured using LPA-SPT (with frame repetition time of 320 ms). The mean (±s.e.m.) mobility (apparent diffusivity, *D*) of clusters in each track-length bin (bin size = 15 frames) is shown. Track length serves as a proxy for cluster size (see text)

The association between the cytoplasmic membrane and other cellular components can in principle affect the local environment of the receptors and, therefore, can potentially affect clustering. The cytoplasmic membrane is physically connected to the cell-wall matrix by various components, including the TolA transmembrane protein that as a part of the Tol–Pal system plays a role in maintaining the integrity of the cell envelope. The Tol–Pal system is also recruited to support the cell division process and has been shown to affect the clustering of chemoreceptors^25^. To further investigate the effect of TolA on chemoreceptor clustering, we constructed the strain *cheA::mYFP* Δ*tolA::kn* (MK13), which carries the tagged CheA but lacks TolA. We found that the prominent effect of the TolA deletion on chemoreceptor clustering is an overall fragmentation of receptor clusters; in the absence of TolA, the clusters were typically smaller but approximately threefold more clusters per cell were observed (Fig. 6a). However, the preference of the receptors to the pole was notable, and could be clearly demonstrated by expressing CheW-X2 in these cells (Fig. 6a and Supplementary Fig. 7). These observations are consistence with those reported in ref. ^22^. Thus, although the Tol system can affect receptor clustering, the TolA protein is not essential for establishing a polar preference.

The cytoplasmic membrane is also associated with the nucleoid through the overlapping processes of transcription, translation and insertion of membrane proteins, termed ‘transertion’^37–39^. In the presence of the antibiotic chloramphenicol (*Cm*), the translation process is arrested, and the nucleoid is condensed^40,41^, although the direct mechanism is still debated^38,39,41^. Here, we found that native receptor clusters that were formed prior to the exposure to *Cm* were mostly stable in the presence of *Cm* (Supplementary Fig. 8A). However, this observation does not preclude the possibility that the formation of new clusters is affected by *Cm*, although such effect cannot be directly tested in *Cm*-sensitive cells. We therefore tested the effect of *Cm* on Tsr(I377P)-mYFP receptor variant, which exhibits considerably reduced tendency for self-association^32^, and therefore may be more dynamic. Exposure to *Cm* for ~20–30 min clearly altered the spatial distribution pattern of these receptors (Fig. 6b). The exposure to *Cm* also led to condensation of the nucleoid within the same time frame, followed by tagging the DNA-binding protein H-NS with mYFP (Fig. 6b and Supplementary Fig. 8B). In contrast, the Tar(head)-mYFP receptors retained their uniform distribution in the presence of *Cm* (Supplementary Fig. 8C), indicating that the observed effect of *Cm* on the Tsr(I377P) receptors is not a general response of membrane proteins. The exposure to *Cm* did not affect the Tsr(I377P)-mYFP receptors when the cells were also transformed with the pBAD33(*Cm*^R^) plasmid that expresses the CAT enzyme, which acetylates *Cm* but does not degrade it (Supplementary Fig. 8D). An exposure to NaCN also inhibited cell growth but did not affect the spatial distribution of the Tsr(I377P)-mYFP receptors (Supplementary Fig. 8E) and, correspondingly, did not  lead to condensation of the nucleoid^42^ (Supplementary Fig. 8F). These observations suggest that the effect of *Cm* on the spatial distribution pattern of the Tsr(I377P) receptors results from its known capacity to condense the nucleoid, which, in turn, affect the membrane environment.

Since, the membrane environment can be expected to differ between the lateral and polar regions, it can lead to distinct behaviors of receptor complexes in those regions, and in particular, to distinct diffusion properties. Indeed, it has been previously demonstrated that within a ~30-min period, polar clusters can randomly move, but lateral clusters cannot^21^. Here, we further investigated the size-dependent mobility of small receptor complexes at the lateral and polar regions at the single-molecule resolution. Cells expressing Tar::mEos2 were examined by localized photoactivation single particle tracking^43^ (LPA-SPT, Methods section). To probe the local membrane environment at the lateral and polar regions, receptor complexes were tracked over short distances (~0.2 µm) and times (10–30 s). The track length of a receptor complex is limited by the number of activated fluorophores within the complex, which, in turn, scale with the total number of tagged receptors in the complex. The track length can therefore provide information about the size of the complexes; tracks of larger complexes will be longer, on average, than tracks of smaller complexes. The mobility of the receptor complexes was generically subdiffusive^43^, and their apparent diffusion coefficient (*D*_app_) decreased monotonically as their size (track length) increased (Fig. 6c). However, the quantitative dependence of the diffusion coefficient on complex size clearly differed between the lateral and polar cell regions; whereas larger complexes tended to have a lower mobility in the lateral region than at the poles (in agreement with ref. ^21^), surprisingly this relationship was opposite for the smaller complexes. Thus, these experiments demonstrate that the local membrane environment of receptor complexes affects their mobility and differs between the lateral and polar regions.

## Discussion

**Fig. 7:**
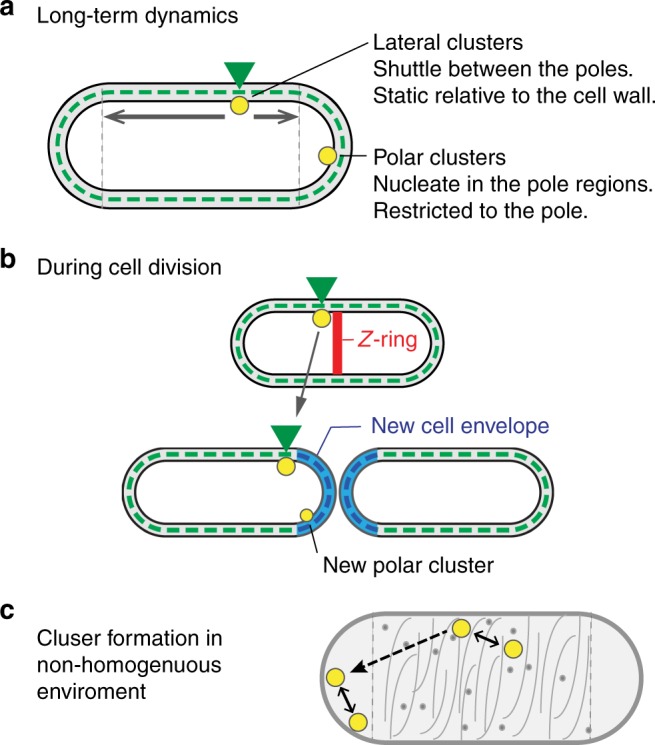
Positioning of receptor clusters. **a** The long-term positional dynamics of clusters. Clusters that nucleate in pole regions remain polar. Clusters that nucleate in the lateral region remain static relative to their local cell-wall matrix (green arrowhead) for many generations, at least in the direction of the long axis of the cell, and thus effectively shuttle between the cell poles. **b** Mid-cell lateral clusters avoid becoming polar during cell division by remaining static relative to their original cell-wall environment and avoid translocation to the new pole region (blue). **c** To the extent that the cell envelope physiology affects the local environment of the receptors, it might be expected that variations are created between the pole and lateral regions. Such variations can potentially affect the basic ‘diffusion and capture’ clustering dynamics by affecting the local mobility and clustering efficiency of receptor complexes and, thus, possibly promote polar preference

By following the long-term positional dynamics of chemoreceptor clusters, we identified two distinct behaviors (Fig. 7a): clusters that nucleated directly at the cell pole during or after cell division remained polar in future generations (polar behavior), and clusters that nucleated at the lateral region mostly remained lateral and as the cell underwent cycles of elongation and division continued to shuttle between the cell poles (lateral behavior). In particular, these dynamics indicates that the lateral clusters does not follow a developmental-like progression, in which lateral clusters eventually become polar; the lateral clusters could transiently locate near the cell poles, but in most cases, lateral clusters avoided trapping in the cell pole and drifted away from the pole in future generations (Fig. 2). Interestingly, the positional dynamics of the lateral clusters obey the dynamics described by Eq. (1) (Fig. 2f and Supplementary Fig. 3), which represents the dynamics expected for a fixed point on an elastic rod that undergoes cycles of stretching and splitting. Since the peptidoglycan matrix is the semi-rigid structural component that physically determines the shape of the cell during elongation and division, lateral clusters must be effectively static relative to this cell-wall matrix for many generations, at least along the long axis of the cell. We also identified a few clusters that effectively split near the boundary between the pole and lateral regions with the resulting two clusters exhibiting distinct behaviors: the cluster closer to the cell tip exhibited polar behavior and remained in the pole, while the cluster that was slightly away from the pole exhibited lateral behavior and drifted away from the pole (Fig. 2c and Supplementary Fig. 3C). Such distinct behaviors of close by clusters are consistent with an underlying sharp boundary, most likely the boundary between the dynamic cell-wall matrix in the lateral region and the inert cell-wall matrix in the pole region.

Underlying the long-term dynamics of clusters is the fact that pre-existing mid-cell clusters tend to avoid trapping in the pole region during cell division (Fig. 2 and Supplementary Fig. 2). This behavior can be explained as follows (Fig. 7b). The future division plane is determined by the positioning of the FtsZ ring. Since chemoreceptor arrays are generally large entities and, as discussed above, static relative to the cell-wall matrix, a pre-existing lateral cluster at the future division plane can potentially physically interfere with the polymerization of FtsZ such that an uninterrupted FtsZ ring can only form next to the receptor arrays. However, because the molecular machinery that creates the new cell pole envelope is generally associated with the FtsZ ring^30,44^ the new cell envelope will be formed between the FtsZ ring and the cluster. Therefore, a mid-cell cluster can become stably polar only if it translocates from its original cell envelope environment to the newly formed cell envelope region. Such translocation might generally depend on the local biophysical conditions during cell division, which might affect cluster mobility, and was evidently infrequent in the experiments presented here (Fig. 2e).

Since most lateral cluster avoid translocation to the poles, the polar preference of the receptors is fundamentally determined by the local nucleation probability and growth rate of clusters in the polar and lateral regions. We indeed find that both the effective nucleation probability of clusters (Fig. 2e) and their growth rate (Fig. 3) were reduced in the lateral region. The local conditions experienced by the receptors in the lateral region are therefore generally less conducive to receptor clustering than those experienced by the receptors in the pole regions. Interestingly, polar preference was clearly enhanced under slower growth conditions induced by poorer medium (Fig. 2e, lower TB content). Notably, the nucleoid is expected to expand under these conditions^41^, which could affect clustering (Fig. 6b). Reduction in lateral clusters under poorer growth conditions is generally consistent with the reduction in lateral clusters in cell cultures at later growth stages (Fig. 1c). Given that even under such slow-growth conditions lateral clusters still avoided translocation to the cell pole regions (Fig. 3e), we suggest that the lateral clusters that emerge at earlier stages are being progressively diluted in the population rather than becoming polar.

The polar preference of the receptors was basically driven by receptor clustering, either with or without CheA/W, with weakly interacting dimers or core complexes showing only weak or no polar preference (Fig. 4a–c). However, weakening part of the connections that holds the native network of core-complexes clearly enhanced their polar bias (Fig. 4d). Weakening the connections between core complexes is expected to affect the assembly process of receptor arrays, reducing their clustering probability, and altering their final structures, presumably rendering them less cohesive. Less efficient clustering can also allow small clusters to diffuse to greater distances without growing in size. These modifications can clearly affect the ‘diffusion and capture’ clustering dynamics^18–20^. Evidently, under these conditions, receptor clustering was specifically reduced in the lateral region (Fig. 4d).

A match between the geometrical structure of receptor complexes and the local curvature of the cytoplasmic membrane can lead to a polar preference^23,24^. However, even receptors in which the orientations of the transmembrane and cytoplasmic domains are uncoupled still exhibit clear polar bias (Fig. 5b, c), indicating that the specific geometrical structure of the receptor trimers is not essential for their polar preference. This observation is consistent with that reported in ref. ^22^, which tested the partition of lateral clustering between the inner and outer sides of bended filamentous cells. As suggested in ref. ^22^, polar preference can also be driven by an entropy-based affinity of receptor complexes toward curved surfaces. This mechanism is indeed more general but still expected to critically depend on the position of the contacts between receptor dimers and the length and flexibility of the dimers. However, receptors missing the vast majority of their potentially flexible part still exhibit a clear polar preference (Fig. 5b). Polar preference was clearly observed even when the head domains of the receptors could interact and, therefore, eliminate their potential entropic contribution (Fig. 5f). Finally, such curvature-based model cannot account for the clear polar bias of TorS sensors (Fig. 5e), which form clusters through direct interactions between the receptor head domains^36^. Taken together, these observations suggest that factors other than membrane curvature contribute to the polar preference of receptor complexes.

The observation that the polar preference of the receptors is directly promoted by their clustering and mostly insensitive to the structural properties of the receptors or their organization in clusters (Figs. 4 and 5) suggest that polar preference may result from factors that affect the basic ‘diffusion and capture’ dynamics underling receptor clustering^18–20^. Possible candidates are the biophysical properties underling the subdiffusive mobility of receptor complexes^43^ or the long-term restricted mobility of the lateral clusters (Fig. 2), which can naturally affect clustering efficiency and long-range mobility of receptor complexes. The direct or indirect associations between the cytoplasmic membrane and various cellular components, such as membrane-bound MreB, the peptidoglycan matrix, or the nucleoid, can potentially affect the local environment of the receptors in the membrane. For example, the disassembly of the MreB network has been shown to affect the mobility of transmembrane proteins^45^. This view is also supported by the observation that factors that affect the association between the membrane and the peptidoglycan matrix (TolA; Fig. 6a) or the nucleoid (*Cm*; Fig. 6b) also affected receptor clustering. Finally, the clustering bias found in bended filamentous cells between the inner and outer sides^22^ can also be consistent with this view, because bending of cells can also lead to asymmetry in the cell envelope environment; for example, due to enhancement of cell-wall components in regions with negative curvature^46^. The association between the cytoplasmic membrane and other cellular components can also lead to distinct environments in the polar and lateral regions of the cell. This view is supported by the observation that the size-dependent mobility of receptor complexes differs between the polar and lateral regions (Fig. 6c)^21^. Under such conditions of distinct dynamical properties of receptor complexes in the polar and lateral environment, a basic diffusion and capture process can possibly lead to polar preference, governed by the competition between local clustering efficiency and long-range diffusion (Fig. 7c).

## Methods

### Bacterial strains and plasmids

Strains and plasmids used in this work are listed in Supplementary Table 1. The *E. coli* strains UU2612, UU2806, UU1607, and VS172 are isogenic derivatives of the parental strain RP437^47^. The *E. coli* strains MK4 (*cheA::myfp*), MK9 (*cheA::myfp cheW-X2*) and MK13 (*cheA::myfp tolA::Kn*) are derivatives of the MG1655(IS1) strain^17^.

### Bacterial colony growth and imaging

Overnight cultures were grown in 1 ml of TB and then diluted 200-fold in fresh growth media containing 20% TB (10 g/L tryptone and 5 g/L NaCl) and 80% M9 minimal media supplemented with 0.1% casamino acids, 1 mM MgCl_2_, 1 mM Na_2_SO_4_, and 1.8 nM thiamine. This ‘standard’ medium was used unless mentioned otherwise. Cultures were grown with agitation for ~2.5 h at 33.5 °C to an OD_600_ of 0.1–0.2. Using a titanium chamber (0.9 ml; 1.8 cm^2^), we cast a 1% agarose gel (based on the standard media). The culture was then again diluted 1:4, and 4 µl were applied to the top of the gel surface and covered with a coverslip. The chamber was then placed in a Nikon Ti microscope equipped with a perfect-focus system, heating stage (set to 32 °C), and ×100 Plan-Fluor objective (1.3 NA). The initial bacterial generation time was ~40–50 min under these conditions. Colonies were followed for up to 6 h with varying frame rates and up to one frame every 4 min, which allowed us to reliably follow individual clusters as they propagated in the colony. The Images were handled in ImageJ and are shown after running 9 × 9 running-average filter. For the images presented in the figures, we set the lower threshold to match the background level and tended to set the upper threshold to match the high intensity pixels. In all cases, both the low and high thresholds were the same across the entire image, or between images if direct comparison is made between them, such as in the case of Figs. 1c and 6b. However, since the intensity difference between clusters can be rather high (~1:100), in cases where it was important to allow clear observation of the smaller clusters, such as the new polar clusters in Fig. 2a–c, the upper threshold was reduced, resulting in apparent image saturation at the positions of larger clusters.

### Measurements of receptor complex mobility (LPA-SPT)

Using photoconvertible fluorescent proteins, LPA-SPT employs a similar principle as standard single-particle tracking methods, but with the difference that photoactivation occurs within ‘hotspots’ on the sample plane generated by diffraction-limited focusing of photoactivation beamlets^43^. This spatially non-uniform photoactivation protocol enables measurement of longer tracks, by photoactivating multiple labeled molecules per cluster, while maintaining a low spatial density of photoactivated clusters.

Strain VS172 was transformed with plasmid pSJAB4 (*tar::mEOS2* on vector pTrc99A). Cells were grown overnight in TB, then inoculated 1:100 in H1 minimal salts medium supplemented with 0.5% glycerol, 1 mM histidine, 1 mM leucine, 1 mM L-methionine, 1 mM threonine, 100 μg/ml ampicillin, 20 μM IPTG, harvested at OD ≈ 0.25, washed twice and suspended in motility buffer (10 mM KPO, 0.1 mM EDTA, 10 mM lactic acid, 1 µM Methionine, pH7) until imaging. For imaging, cells were immobilized by anti-flagellar antibody (a gift of H.C. Berg, Harvard University) on a round coverslip (no.1), and incubated in a stainless steel flow cell under motility buffer flow at 400 μL/min. A square grid of diffraction-limited photoactivation foci was generated at the sample plane using a microlens array in the optical path, to switch mEos2 fluorophores from the green-emitting (inactive) to red-emitting (active) state for subsequent single-particle tracking. Photoactivation of mEOS2 fluorophores at these hotspots occurred through photoactivation-acquisition cycles in which delivery of a short (1 ms) photoactivation pulse from a 405 nm laser was followed by an acquisition sequence of 249 image frames (at *t*_f_ = 320 ms intervals, with 30 ms exposure to wide-field laser excitation delivered by a 568 nm laser). This cycle was repeated four times per field of view, and under these conditions we typically obtain ~500 tracks per field of view (after filtering for those with track length >=5 frames) for subsequent analysis. Greater than 99.99% of photoactivated clusters were bleached within each 250-frame cycle so that before each 405 nm pulse, no mEos2 fluorophores were visible in the red emission channel. After each pulse, a few Tar-mEos2 clusters appeared near each of 25 photoactivation hotspots within the field of view, as the mEOS2 labels were switched to the red-emitting state by 405 nm irradiation. Focal drift in the *z*-direction was minimized by closed-loop feedback (Perfect Focus System, Nikon). This 4-cycle LPA-SPT imaging sequence was repeated across many fields of view, sampled at ~250 μm intervals across the field of coverslip-attached cells.

Single-particle tracks were extracted from LPA-SPT image sequences using the u-track code, integrated into custom MATLAB scripts to automate data handling. A bright-field image was acquired in each field of view, and segmented to extract binary images of the cell body which were used to classify tacks into lateral and polar categories (we defined the polar region as the set of points within a distance *W*/2 from the pole, measured along the long axis of the cell, where *W* is the width of the cell. For each track we identified the cell to which it belongs, as well as its location along the cell long axis. By tuning the energy of the photoactivation pulse (i.e. pulse intensity times pulse duration) LPA-SPT allows multiple fluorophore labels to be photoactivated within tracked clusters, and the number *n* of activated fluorophores follows a binomial distribution with a mean <*n*> = *Np* that increases with the cluster size *N* (i.e. the number of fluorophores in the cluster) and the probability *p* that each of *N* fluorophores is switched on by the photoactivation pulse. After photoactivation, the length of tracks with *n* initially active labels is determined by the first-passage time t* at which the number of molecules in the emitting state drops from its initial value of *n* to below the detection threshold n* (0 < n* < 1 under our experimental conditions). The distribution of t* depends on details of the bleaching/blinking statistics, but a generic feature of first-passage times is that the expectation value *E*[t*] is an increasing function of the difference between the initial value and the threshold, *n* − n* (≈*n* under our experimental conditions). Thus, *E*[t*] must increase with <*n*> = *Np* and the track length t*/*t*_f_ carries information about the cluster size *N*.

### Soft agar chemotaxis assay

A total of 140 mm plates were filled with Tryptone Broth medium containing 0.25% Bacto-agar and allowed to solidify. One microliter of each cell suspension was inoculated in the agar in a confined spot and plates were incubated for 10 h at 30 °C, after which a dark-field image of the plate was taken. The expansion of a colony in such plates is driven by chemotactic responses to the gradients formed in the plate by the consumption of amino acids, primarily serine, and aspartate^28^.

## Electronic supplementary material

Supplementary Information

## Data Availability

The data that support the findings of this study are available from the corresponding author upon reasonable request.
