# Supplementary material for: Long-term positioning and polar preference of chemoreceptor clusters in E. coli
Source: Nat Commun. 2018 Oct 25;9:4444. doi: 10.1038/s41467-018-06835-5 (PMC6202326; doi:10.1038/s41467-018-06835-5)
Supplement: Supplementary file 1 — Supplementary Information [file 41467_2018_6835_MOESM1_ESM.pdf]

Supplementary Information

Supplementary Figure 1

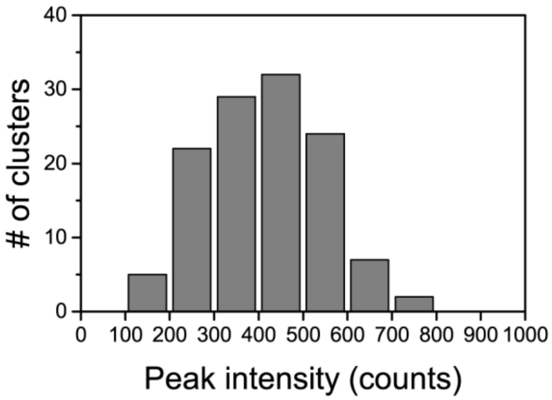

Distribution of cluster peak intensity in IL2 cells (containing chromosomal cassette of 240 TetR binding sites) expressing TetR-mYFP (induced by $1\text{-}2{\cdot}10^{-4}$ % arabinose). The saturated intensity, which corresponds to ~240 mYFP molecules, is approximately 500-700. Therefore, the minimally detected chemoreceptor cluster, which under the same conditions has an intensity of 50-70, approximately contains 17-33 mYFP molecules, or 8-16 receptor core units.

Supplementary Figure 2

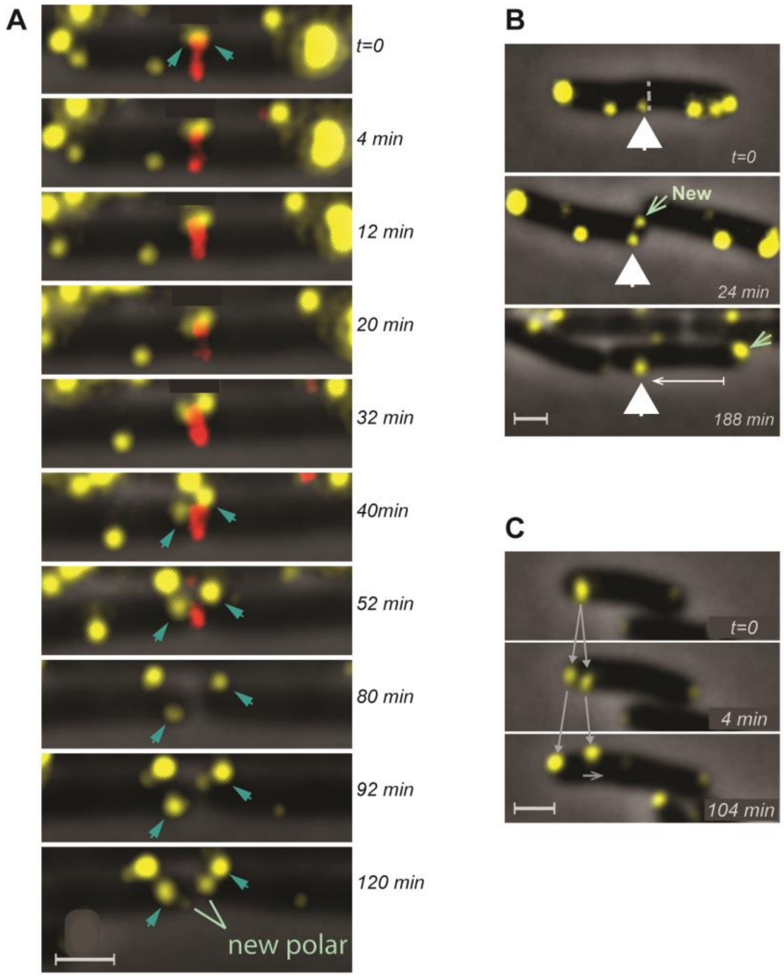

Positional dynamics of chemoreceptor clusters in *cheA::mYFP* (MK4) cells. (**A**) In these examples cells are expressing FtsZ-mCherry (induced by 0.005% arabinose). At t=0 there is an apparent single cluster (arrows) directly at the future cell division plan, marked by the FtsZ-mCherry fluorescence (red). However, at later times, the cluster split into two clusters positioned beside the division plan, each on different side of the Z-ring. These clusters then become polar after cell division, but at later times new clusters form directly at the new poles. (**B**) Additional example in cells that do not contain the tagged FtsZ. In this example, again, a single cluster (arrows) near the cell division plane (dashed line), after division near the polar region, while additional new cluster appears during cell division at the same pole. However, later on, the former cluster drift away from the pole, while the new cluster remains polar. (**C**) Single cluster near the cell the pole effectively split into two clusters. Later on, the cluster closer slightly further from the pole drift away from the pole while to the cluster closer to the pole remains at the pole. Scale bars corresponds to 1 μm.

Supplementary Figure 3

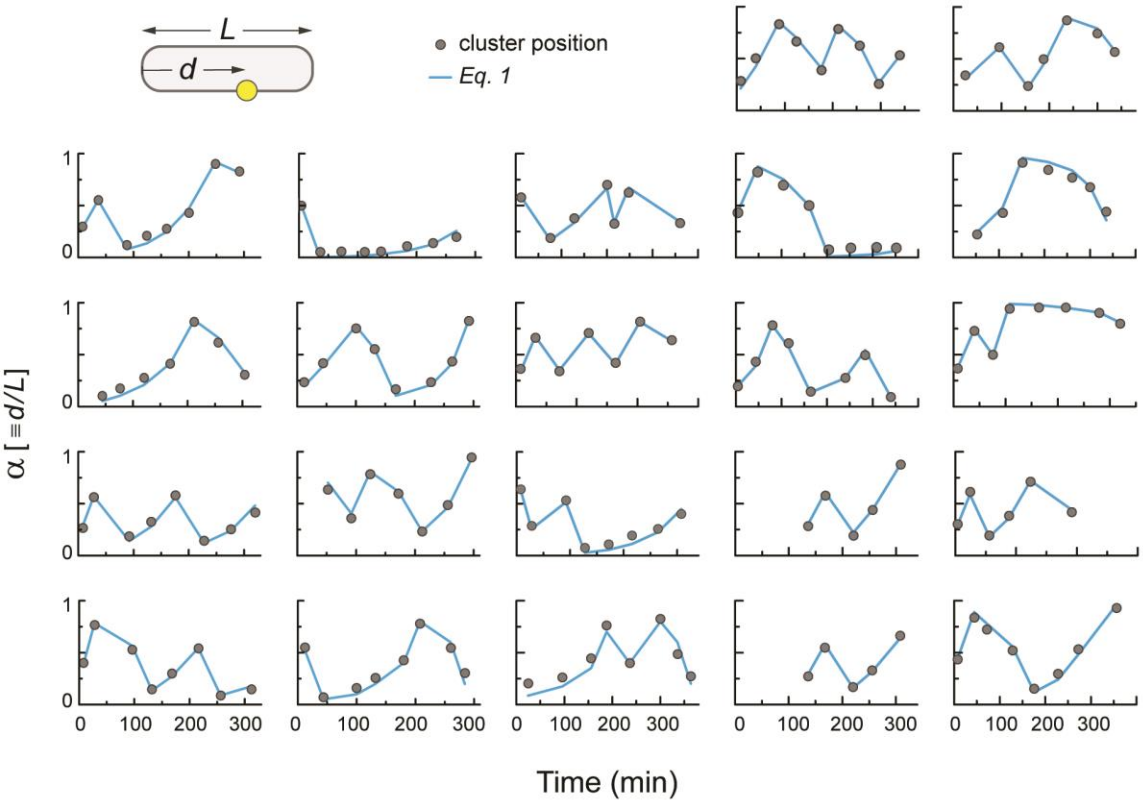

Positional dynamics of lateral clusters. The relative position of a clusters (α) —defined as the distance of a cluster from a certain cell pole divided by the length of the cell— is plotted as a function of time (gray circles). Each plot represents the trajectory of a single cluster whose position was sampled once every cell cycle, soon after cell division. The blue lines were plotted by iterating Eq. 1 (see text). Total of 22 clusters from 8 independent experiments were followed, each for several generations.

Supplementary Figure 4

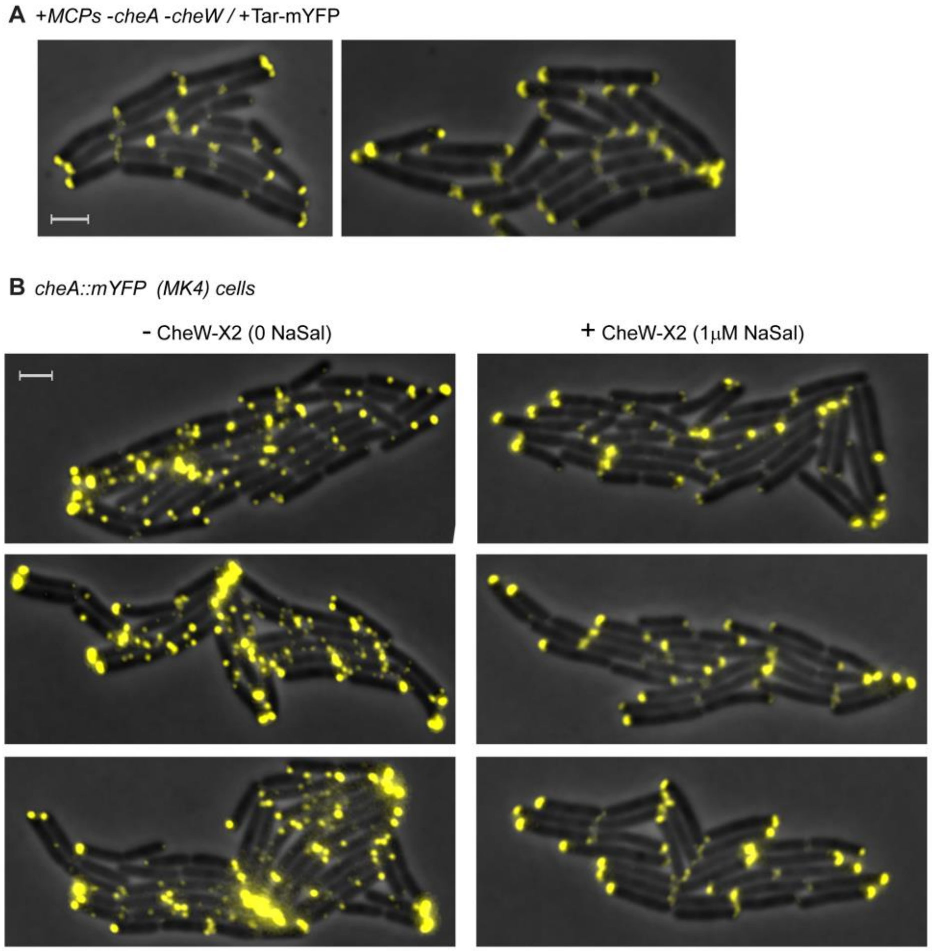

Modified receptor arrays. (**A**) Tar-mYFP expressed in addition to the native receptors in cells lacking CheA and CheW (UU1607). (**B**) Colonies of *cheA::mYFP* cells (MK4) with or without the addition of CheW-X2 (0 or 1 μM NaSal, respectively). Scale bars corresponds to 2 μm.

Supplementary Figure 5

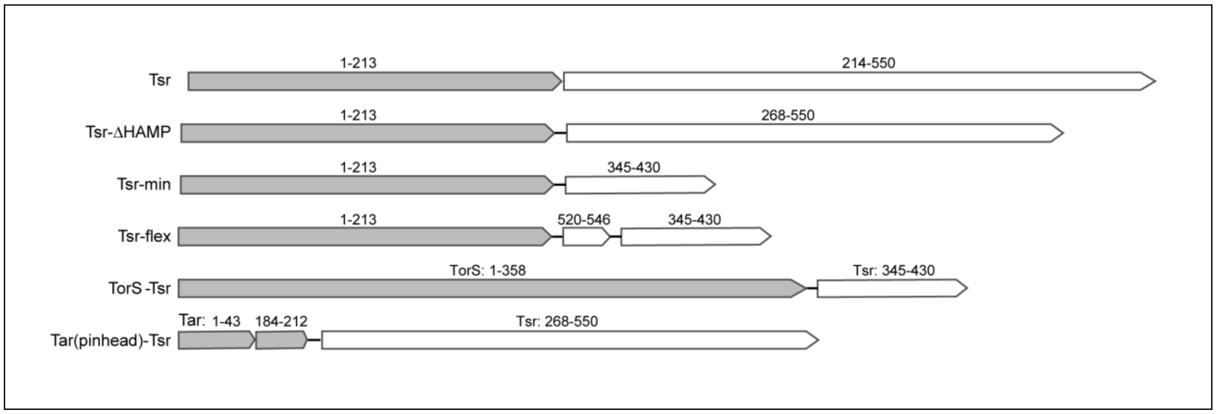

Schematic description of the receptor constructs tested in Figure 4. Regions encoding for the periplasmic-transmembrane domains are shown in gray and those encoding for the cytoplasmic domains are shown in white.

Supplementary Figure 6

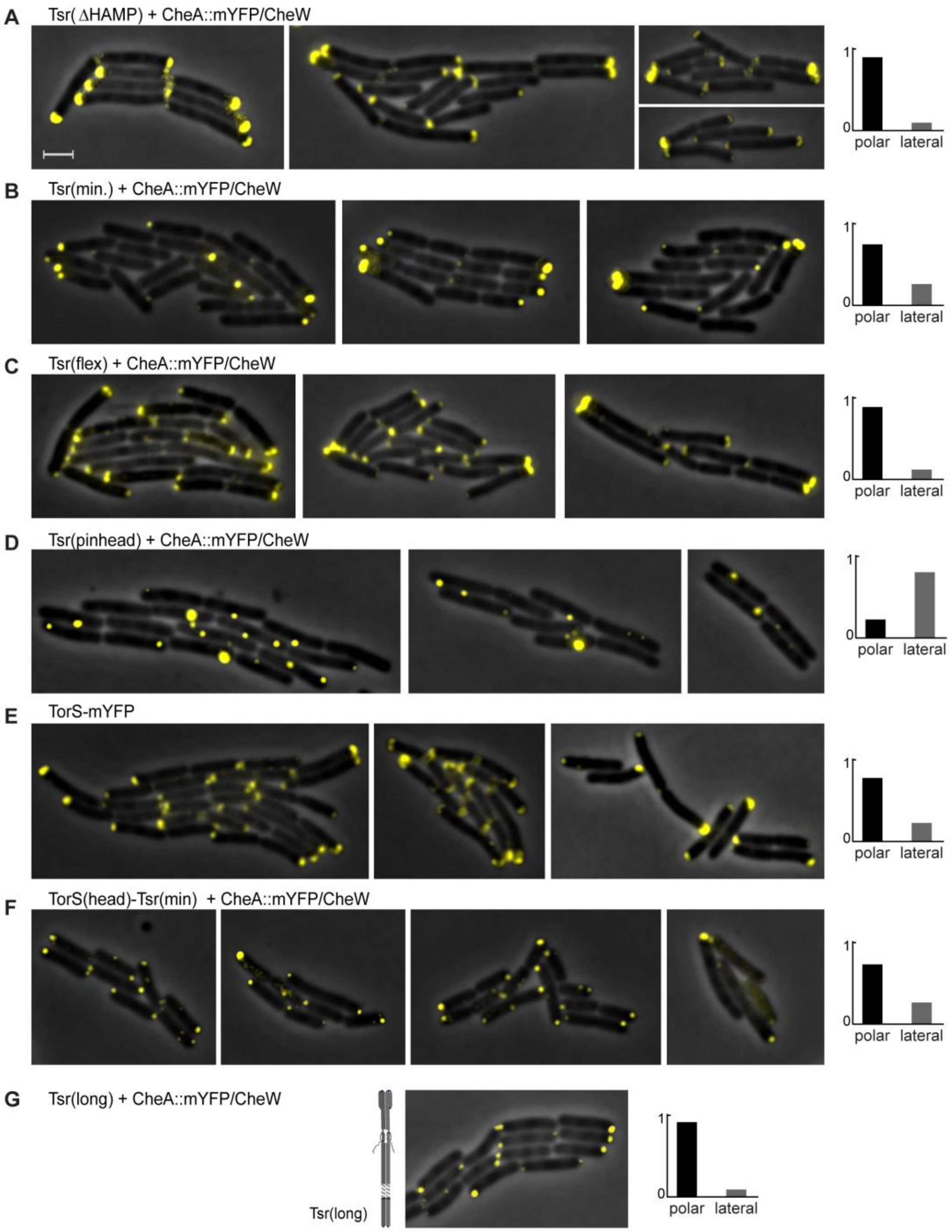

Clustering of modified receptors. (**A-D** and **F-G**) Fluorescence images of *cheA*$^{-}$ *cheW*$^{-}$ *MCPs*$^{-}$ (UU2806) cells expressing each receptor variant (pRR53 plasmid, induced with 100-200 µM IPTG) together with CheA::mYFP and CheW (pKG110 plasmid, induced with 0.3 µM NaSal). Receptor variants are specified in Fig. S5 and Table S1. (**E**) Images of TorS-mYFP expressed in MG1655 cells (ptrc99A plasmid, induced with 10 µM IPTG). Also shown for each receptor variant is the fraction of polar and lateral clusters found in these (and similar) colonies (a total of 208, 148, 88, 206, 169, 191 and 78 clusters analyzed in parts A through G, respectively). Notably, in addition to the clear bias in the counts of polar and lateral cluster, the clusters were generically larger in the pole regions than in the lateral region. Scale bar corresponds to 2 µm, throughout.

Supplementary Figure 7

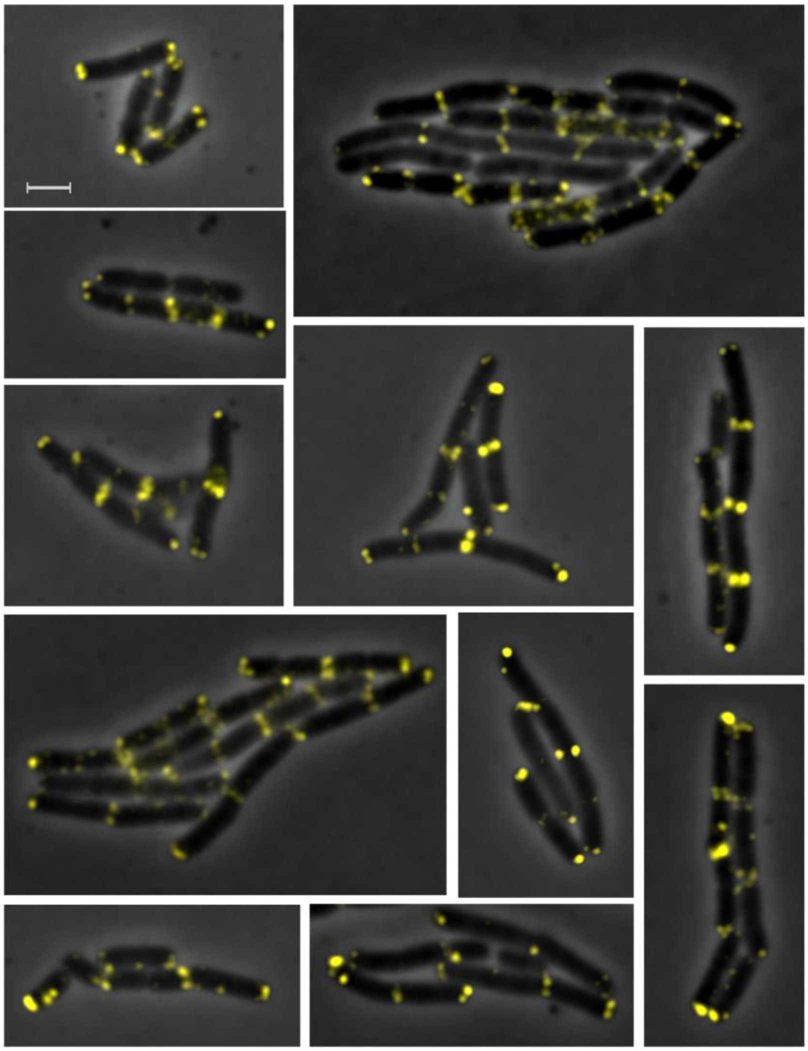

Additional examples of MK13 (TolA^{-}) cells demonstrating clear polar bias when CheW-X2 is also expressed from a plasmid (pAV305) using 1 µM NaSal. Scale bar corresponds to 2 µm, throughout.

Supplementary Figure 8

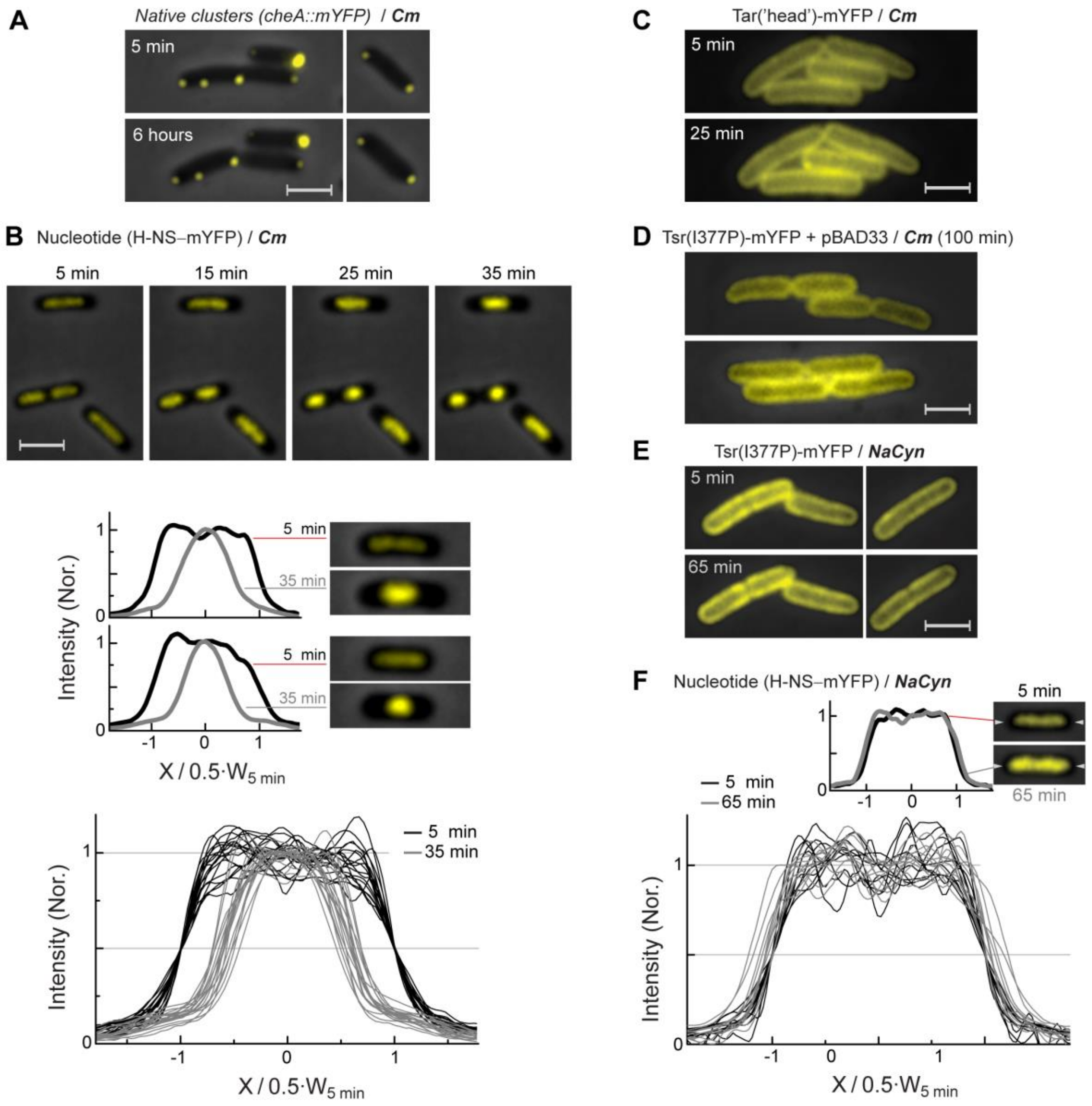

Effect of *Cm* on clustering and nucleoid condensation. (**A**) *cheA::mYFP* (MK4) cells imaged 5 min and 6 hours after exposure to *Cm* (20 µm/ml). (**B**) *cheA*$^-$ *cheW*$^-$ *MCPs*$^-$ (UU2806) cells expressing H-NS-mYFP (pAV357, induced by 2 µM IPTG) and imaged at various times after exposure to *Cm* (20 µm/ml). *Upper panel* – progression of nucleoid over time. *Middle panel* – examples of two cells including their images and the corresponding intensity profiles along the cell. *Lower panel* – intensity profiles of 18 cells 5 min and 35 min after exposure to *Cm*. For each cell, the width of the profiles at both time points were normalized to the width at 5 min. (**C**) *cheA*$^-$ *cheW*$^-$ *MCPs*$^-$ (UU2806) cells expressing Tar(head)-mYFP imaged 5 and 25 min after exposure to *Cm* (20 µm/ml). (**D**) *cheA*$^-$ *cheW*$^-$ *MCPs*$^-$ (UU2806) harboring 'empty' pBAD33 plasmid and expressing Tsr(I377P)-mYFP receptors growing in the presence of *Cm* (20 µm/ml). (**E**) *cheA*$^-$ *cheW*$^-$ *MCPs*$^-$ (UU2806) cells expressing Tsr(I377P)-mYFP receptors imaged 5 and 65 min after exposure to NaCyn (1 mM). (**F**) Similar to (B) except that the cells were exposed to NaCyn (1 mM). Scale bars corresponds to 2 µm.

Supplementary Note 1 – Derivation of equation (1)

As mentioned in the text, we assume that lateral clusters:

(i) Are fixed relative to the local cell-wall environment, at least, along the long axis of the cell.

(ii) Remain within the dynamic region of the cell envelope that undergoes elongation.

Thus, we consider a certain point on a rod that undergoes cycles of homogeneous stretching (exponential growth) and then division.

In an exponentially growing rod, the distance of an arbitrary fixed point from the left (l) or right (r) pole is given by

$$L_{l,r}(t) = L_{l,r}^{0} \cdot \exp(\gamma \cdot t)$$

and

$$\alpha = \frac{L_l}{L_r + L_l} = \frac{1}{1 + L_r/L_l}$$

Therefore, during cell elongation, since the ratio L_l/L_r remains constant, the relative position α also remains constant.

Upon cell division, the distance of the point from the closest pole is unchanged, but the length of the cell is halved. Therefore, if α was smaller than 1/2 prior to cell division, it doubles upon cell division, or $\alpha_n = 2 \cdot \alpha_{n-1}$. Alternatively, if α was larger than 1/2 prior to cell division, the same is true relative to the opposite (closer) pole, and therefore $(1 - \alpha)$ doubles, or $\alpha_n = 2 \cdot \alpha_{n-1} - 1$.

Taken together, after each cycle (*n*) of stretching and division

$$Eq.(1) \qquad \alpha_n = 2 \cdot \alpha_{n-1} \;\; Modulo\ 1$$

Supplementary Table 1 – Strains and plasmids

Strain	Genotype	reference
	**Derivatives of MG1655**	
VF6	*MG1655*	1
MK4	*cheA(1-146)-myfp-cheA(147-end)*	This work
MK9	*cheA(1-146)-myfp-cheA(147-end) cheW(R117D, F122S)*	This work
MK13	*cheA(1-146)-myfp-cheA(147-end) ΔtolA::Kn*	This work
	**Derivatives of RP437**	
uu2612	*Δ(tar-tap) Δ(aer) Δ(tsr) Δ(trg)*	2
uu2806	*Δ(cheA-cheW-tar-tap-cheR-cheB-cheY-cheZ)Δ(tsr) Δ(aer) Δ(trg)*	3
uu1607	*Δ(cheA-cheW)*	4
VS172	Δ(*tar-cheZ*)	5

Plasmid		reference
pRR48		6
pKG116		7
pAV28	*pTrc99A / tar (0-279)-myfp*	8
pAV30	*pTrc99A / tsr(I377P)-myfp*	8
pES42	*pTrc99A / torS-myfp*	9
pSJAB4	*pTrc99A / tar::mEOS2*	10
pAV309	*pBAD33 / ftsZ-mcherry*	This work
pAV313	*pRR48 / tsr Δ(214-267)Ω(GGGSGG)*	This work
pAV316	*pRR48 / tsr (1-213)-(GGGSGG)-(345-430)*	This work
pAV317	*pRR48 / tsr (1-213)-(GGGSGG)-(520-546)-(345-430)*	This work
pAV326	*pRR48 / torS(1-358)-(GGGSGG)-Tsr (345-430)*	This work
pAV329	*pRR48 / tar (0-43)-Tar(184-212)-(GGGSGG)-Tsr (268-550)*	This work
pAV357	*pRR48 / H-NS-mYFP*	This work
pPM24	*pKG116 / cheW*	Parkinson lab
pAV305	*pKG116 / cheW-X2 (R117D F122S)*	This work
pAV214	*pKG116 / Tar(QEQE)[1-520]-myfp*	This work
pAV295	*pKG116 / cheA(1-146)-myfp-cheA(147-654)-cheW*	This work
pTsrH18.8	*pKG116 / long Tsr derivative*	11
pAV311	*pKG116 / cheA(1-146)-myfp-cheA(147-654)-cheW (R117D, F122S)*	This work

Supplementary References

1 Frank, V., Piñas, G. E., Cohen, H., Parkinson, J. S. & Vaknin, A. Networked chemoreceptors benefit bacterial chemotaxis performance. *mBio* **7** (2016).

2 Zhou, Q., Ames, P. & Parkinson, J. S. Biphasic control logic of HAMP domain signalling in the *Escherichia coli* serine chemoreceptor. *Mol. Microbiol.* **80**, 596-611 (2011).

3 Piñas, G. E., Frank, V., Vaknin, A. & Parkinson, J. S. The source of high signal cooperativity in bacterial chemosensory arrays. *Proc. Natl. Acad. Sci.* **113**, 3335-3340 (2016).

4 Zhang, P., Khursigara, C. M., Hartnell, L. M. & Subramaniam, S. Direct visualization of *Escherichia coli* chemotaxis receptor arrays using cryo-electron microscopy. *Proc. Natl. Acad. Sci. USA* **104** (2007).

5 Kentner, D., Thiem, S., Hildenbeutel, M. & Sourjik, V. Determinants of chemorecptor cluster formation in *Escherichia coli*. *Mol. Microbiol.* **61**, 407-417 (2006).

6 Studdert, C. A. & Parkinson, J. S. Insights into the organization and dynamics of bacterial chemoreceptor clusters through in vivo crosslinking studies. *Proc. Natl. Acad. Sci. USA* **102** (2005).

7 Gosink, K. K., del Carmen Burón-Barral, M. & Parkinson, J. S. Signaling interactions between the aerotaxis transducer Aer and heterologous chemoreceptors in *Escherichia coli*. *J. Bacteriol.* **188**, 3487-3493 (2006).

8 Vaknin, A. & Berg, H. C. Osmotic stress mechanically perturbs chemoreceptors in *Escherichia coli*. *Proc. Natl. Acad. Sci. USA* **103**, 592-596 (2006).

9 Sommer, E., Koler, M., Frank, V., Sourjik, V. & Vaknin, A. The sensory histidine kinases TorS and EvgS tend to form clusters in *Escherichia coli* Cells. *PLoS ONE* **8**, e77708 (2013).

10 Solari, J., Anquez, F., Scherer, K. & Shimizu, T. S. Bacterial chemoreceptor imaging at high spatio-temporal resolution using photoconvertible fluorescent proteins. *Meth. Mol. Biol.* **1729**, 203-231 (2018).

11 Herrera, S. M. K., Frank, V., Massazza, D. A., Vaknin, A. & Studdert, C. A. Bacterial chemoreceptors of different length classes signal independently. *Mol. Microbiol.* **93**, 814 (2014).
